# Modelling of Electrowetting-Induced Droplet Detachment and Jumping over Topographically Micro-Structured Surfaces

**DOI:** 10.3390/mi12060592

**Published:** 2021-05-21

**Authors:** Alexandros G. Sourais, Athanasios G. Papathanasiou

**Affiliations:** School of Chemical Engineering, National Technical University of Athens, 15780 Athens, Greece; alexandrossourais@mail.ntua.gr

**Keywords:** electrowetting, droplet detachment, micro-structured surfaces, numerical modelling

## Abstract

Detachment and jumping of liquid droplets over solid surfaces under electrowetting actuation are of fundamental interest in many microfluidic and heat transfer applications. In this study we demonstrate the potential capabilities of our continuum-level, sharp-interface modelling approach, which overcomes some important limitations of convectional hydrodynamic models, when simulating droplet detachment and jumping dynamics over flat and micro-structured surfaces. Preliminary calculations reveal a considerable connection between substrate micro-topography and energy efficiency of the process. The latter results could be extended to the optimal design of micro-structured solid surfaces for electrowetting-induced droplet removal in ambient conditions.

## 1. Introduction

Active manipulation of liquid droplets, without the use of moving mechanical parts, is important in a variety of microfluidic applications, ranging from biological/chemical analysis [1] (rapid tests), to the development of devices [2,3] (liquid lenses, micro-cameras, displays, etc.) and self-cleaning surfaces [4]. Especially, the process of droplet detachment and removal from a solid substrate is essential in practical heat transfer applications where condensation and coalescence of droplets can lead to increased thermal resistance [5,6,7,8,9]. Some of the active methods that have been proposed for droplet detachment utilize air flow [10,11], electrostatic forces [12], electrowetting actuation [13,14,15], etc. Among these methods, electrowetting shows some clear advantages such as low energy consumption (takes effect at significantly low voltage), large contact angle variations, fast response time and high reversibility.

In a typical electrowetting-on-dielectric (EWOD) setup, the wettability increases and a conductive droplet spreads under the effect of the electrical force concentrated at the three-phase contact line (TPL). The droplet detachment can be induced by instantly switching the applied voltage off; then the macroscopic contact angle immediately restores back and the excess surface energy can be converted to kinetic energy, driving the droplet to detach. Alternatively, as recently suggested [16,17] it is possible to manipulate, in a similar but more direct and effective way, a non-conductive droplet, surrounded by a conductive fluid, using reversed electrowetting (REW). In this case the application of the voltage increases the wettability of the conductive surrounding medium, leading directly to the de-wetting and, finally, the detachment of the non-conductive droplet.

Apart from experimental studies, it is important to investigate the process using numerical tools, in order to gain more insight into the droplet dynamics and critical parameters affecting the droplet detachment. A great amount of work can be found in the literature covering numerical modelling of droplet detachment under electrowetting actuation [17,18,19,20,21]. Cavalli et al. [18] used a convectional hydrodynamic model with a relaxation factor to recover the no-slip boundary condition as well as a critical condition for the manual disconnection of the contact line. Raman et al. [19] implemented a three-dimensional high-density ratio multiphase lattice Boltzmann method (LB), employing two particle distribution functions to recover incompressible Navier–Stokes equations. Merdasi et al. [21] suggested the idea of enhancing the droplet dynamics of detachment under electrowetting actuation by introducing macroscopically a topographic heterogeneity on the solid substrate. They developed a VOF-CSF (volume of fluid-continuum surface force) hydrodynamic numerical model with no-slip and contact angle boundary conditions, considering electrical, capillary and contact line friction forces. In brief, the main goals of the previous numerical studies regarding electrowetting-induced droplet detachment and jumping, is to develop a sufficiently accurate model, in order to capture the critical parameters affecting droplet dynamics and energy efficiency. The current numerical studies are limited to systems where the solid substrates are treated, at least microscopically, as perfectly smooth and the superhydrophobic behavior is achieved using oil as a surrounding medium. However, this is not the case for many practical applications. For example, in heat transfer applications, the droplet removal must be performed in ambient conditions, using geometrically or chemically textured hydrophobic surfaces combined with an actuation method [8,9]. Furthermore, Cavali et al. [18] suggested the use of micro-textured surfaces for improving the energy efficiency of the droplet jumping process. Thus, a need arises for numerical modelling of electrowetting induced droplet detachment and jumping over micro-structured surfaces.

Convectional hydrodynamic models used for simulating droplet detachment suffer from some important drawbacks. The explicit application of a no-slip boundary condition for the velocity creates a stress singularity and limits the movement of the contact line (TPL). Tracking the movement of the TPL is extremely important during the de-wetting of the droplet. A workaround for this problem is to use a so-called relaxation to a slip boundary condition. The contact line, in this case, is a moving boundary and an additional theoretical correlation between the velocity and the dynamic contact angle must be used, such as the Cox relation [22]. Although this formulation helps in removing the singularity, it cannot be applied to micro-structured surfaces where multiple, unknown contact lines are present. Alternatively, diffuse-interface formulations, such as VOFs and phase-field method, regularize the stress singularity by using a fixed computational mesh and implicit functions to represent different phases. These methods can be used successfully for simulating multiple contact lines on complex surfaces, but exhibit some drawbacks compared to sharp-interface, hydrodynamic formulations such as higher computational cost (need for denser meshes), numerical diffusion, limited accuracy for high deformations and large thickness of the diffuse interface. Lattice Boltzmann (LB) methods, on the other hand, are inappropriate for simulating real size droplets due to their high computational cost.

Recently it was presented by our group a novel, continuum-level, sharp-interface modelling approach, which has been proven particularly efficient for simulating wetting phenomena, especially for complex (geometrically or chemically textured) solid surfaces [23,24,25,26]. In this work, we demonstrate that with this modelling approach it is possible, probably for the first time, to study the effects of substrate microtopography and the resulting wetting states on critical conditions for detachment and droplet jumping dynamics in ambient conditions.

## 2. Materials and Methods 

### 2.1. Hydrodynamic Model

**Figure 1:**
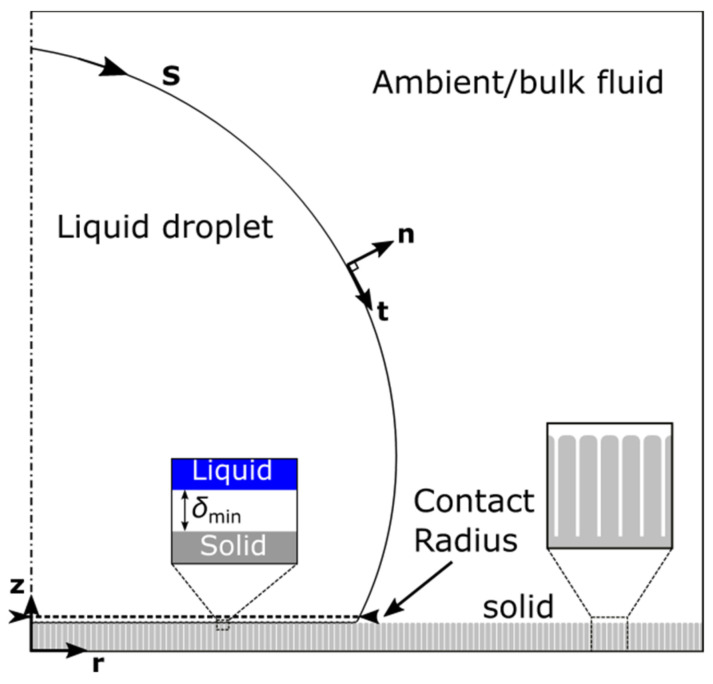
Illustration of the de-wetting/jumping setup of an axisymmetric droplet surrounded by a bulk fluid (air or oil) on a structured solid substrate.

The dynamics of droplet de-wetting, detachment and jumping, are governed by the Navier–Stokes equations of mass and momentum conservation [27] (see Figure 1):

(1) $$\rho \left(\frac{\partial u}{\partial t}+u\cdot \nabla u\right)=-\nabla p+\mu \nabla^{2}u+u,\;\;\begin{array}{c} \nabla \cdot u=0,\; \end{array}$$

where, ρ is the fluid density, μ is the viscosity, $u=(u_{r},u_{z})$ is the velocity field, p, is the pressure and $G\equiv -ge_{z}$ is the gravitational force.

In our formulation the main limitations arising in continuum-level modelling can be overcome by treating the liquid–ambient and the liquid–liquid interface in a unified context. This can be achieved with an extension of the stress balance equation to govern the entire droplet interface. The liquid–solid interactions are described as an effective pressure term, $p^{LS}$, which is introduced in the normal component of the stress balance equation:

(2) $$\begin{array}{c} {\tau_{nn}|}_{ambient}-{\tau_{nn}|}_{liquid}+\Delta p-\gamma_{LA}C-p^{LS}=0,\;at\;S,\; \end{array}$$

where, Δp, is the pressure jump across the liquid–ambient interface, $\gamma_{LA}$, is the liquid–ambient interfacial tension, C, is the local mean curvature and *S* denotes the droplet’s (liquid–ambient or liquid–liquid) interface. The Derjaguin pressure (also called disjoining pressure for liquid films), $p^{LS}$, is commonly defined as the pressure in excess of the bulk pressure that is generated in a thin liquid film between two parallel plates. The latter can be attractive (negative Derjaguin pressure) or repulsive (positive Derjaguin pressure) [25]. The Derjaguin pressure is generated by the following Lennard-Jones type of potential:

(3) $$\begin{array}{c} \frac{R_{0}}{\gamma_{LA}}p^{LS}=w^{LS}\left[{\left(\frac{\sigma }{\frac{\delta }{R_{0}}+\varepsilon }\right)}^{C_{1}}-{\left(\frac{\sigma }{\frac{\delta }{R_{0}}+\varepsilon }\right)}^{C_{2}}\right]\; \end{array}$$

where, $R_{0}$, is a characteristic length defined as the radius of a spherical droplet with the same liquid volume and $\sigma ,\varepsilon ,\;C_{1},\;C_{2}$ are model parameters (see [24,25]). The wettability parameter, $w^{LS}$, can be connected with the Young’s contact angle, $\theta_{Y}$, through Young’s–Dupre equation:

(4) $$\begin{array}{c} w^{LS}=\frac{\left(C_{1}-1\right)\left(C_{1}-1\right)\left(1+cos\theta_{Y}\right)\;}{\sigma \left(C_{1}-C_{2}\right)}\; \end{array}$$

The nature of interactions (repulsive or attractive) is determined by the vertical distance, δ, between the liquid interface and the solid substrate. In general, for non-flat surfaces, we define, δ, as the Euclidean distance from the solid boundary.

The above approach requires that the liquid and solid phases are always separated by a thin intermediate layer with thickness, $\delta_{min}$ (Figure 1). The wettability of the solid material (i.e., Young’s angle) in this framework would emerge naturally as the result of the combined action of the surface tension and liquid–solid interactions. The thickness,$\;\delta_{min}$, is a model parameter and is chosen to be sufficiently small as compared to other system dimensions. For a detailed description and theoretical establishment of the model, including values of the model parameters, see [24,25].

In order to efficiently account for the movement of the droplet interface (the advection of the droplet interface by the velocity field) the following kinematic boundary condition across liquid–ambient interface is solved:

(5) $$\begin{array}{c} \left(u_{mesh}-u\right)\cdot n=0,\;at\;S,\; \end{array}$$

where, $u_{mesh}$, is the velocity of the moving mesh at the interface. The above model is implemented in the commercial software package, COMSOL Multiphysics^®^.

The modification of the wettability (by applying and then removing the voltage) is indirectly incorporated as an instant change in solid material’s wettability ($w^{LS}$ parameter) using a step function. Alternatively, it is possible to couple the modified hydrodynamics equations with the equations of electrostatics (Gauss law) by introducing an electrostatic pressure term in the stress balance equation across the droplet interface, as explained in [26].

### 2.2. Energy Calculations

The critical energy condition which must be, at least, fulfilled to achieve droplet jumping is:

(6) $$\begin{array}{c} \Delta {Ε}_{ex}=E_{ON}-E_{OFF}-\Delta {Ε}_{net}>0,\; \end{array}$$

where $E_{ON}$ is the surface free energy of the stable equilibrium droplet when voltage is ON, $E_{OFF}$ is the surface free energy of the stable equilibrium state when voltage is OFF and $\Delta {Ε}_{net}$ is the net free energy of adhesion (voltage OFF). The energy difference, $\Delta {Ε}_{ex}$, is, actually, the available excess surface energy after removing the net free energy of adhesion. The net free energy of adhesion, $\Delta {Ε}_{net}$, is the reversible work required to separate a sessile drop from a smooth solid surface, to form a free sphere [28,29]. In general, the surface free energy is defined as:

(7) $$\begin{array}{c} E=\gamma_{LS}A_{LS}+\gamma_{SA}A_{SA}+\gamma_{LA}A_{LA},\; \end{array}$$

where $\gamma_{LS},\;\;\gamma_{SA},\;\;\gamma_{LA}$ is the liquid–solid, solid–ambient and liquid–ambient interfacial tension and $A_{LS},\;\;A_{SA},\;\;A_{LA}\;$are the liquid–solid, solid–ambient and liquid–ambient interfacial area, respectively. At equilibrium the interfacial tensions are connected with the Young’s contact angle, $\theta_{Y},$ through the Young’s equation:

(8) $$\begin{array}{c} \gamma_{SA}=\gamma_{LS}+\gamma_{LA}cos\theta_{Y} \end{array}$$

The net free energy of adhesion is given by [28]:

(9) $$\begin{array}{c} \Delta {Ε}_{net}=\pi R_{c}^{2}\gamma_{LA}\left[{\left(\frac{2a_{eff}}{sin\theta_{eff}}\right)}^{2/3}-a_{eff}\right],\; \end{array}$$

where, $R_{c}$, is the contact radius (the radius of the solid–liquid interface), $\theta_{a}$, is the droplet’s effective (macroscopic) contact angle for the non-actuated state and $a_{eff}=\frac{2}{1+cos\theta_{eff}}-cos\theta_{eff}$ is the effective area. We note that, in Equation (9), we treat the micro-structured surfaces as smooth solids, by replacing the Young’s contact angle in the original formulation with the value of the effective contact angle.

Combining the Equations (6)–(9) the available excess energy is computed as [30]:

(10) $$\Delta {Ε}_{ex}=\gamma_{LA}[(A_{\mathrm{LA},\mathrm{ON}}-A_{\mathrm{LA},\mathrm{OFF}})-(A_{\mathrm{LS},\mathrm{ON}}\cos\theta_{Y,\mathrm{ON}}-A_{\mathrm{LS},\mathrm{OFF}}\cos\theta_{Y,\mathrm{OFF}})\;-\pi R_{c}^{2}\left[{\left(\frac{2a_{eff}}{sin\theta_{eff}}\right)}^{\frac{2}{3}}-a_{eff}\right]]$$

The total energy dissipation can be quantified by calculating the viscous dissipation function in axisymmetric form, φ [31]:

(11) $$\begin{array}{c} \varphi \equiv \tau :\nabla u=2\mu [{\left(\frac{\partial u_{r}}{\partial r}\right)}^{2}+{\left(\frac{u_{r}}{r}\right)}^{2}+{\left(\frac{\partial u_{z}}{\partial z}\right)}^{2}+\frac{1}{2}{\left(\frac{\partial u_{z}}{\partial r}+\frac{\partial u_{r}}{\partial z}\right)}^{2}] \end{array}$$

and integrating over time and the entire computational domain Ω:

(12) $$\begin{array}{c} E_{d}=\underset{t}{\int }\underset{\Omega }{\int }\varphi \;d\Omega dt \end{array}$$

In Equation (11), τ:∇u is the double dot product operation, :, between the stress tensor, τ, and the strain-rate tensor, ∇u.

The kinetic energy of the droplet is:

(13) $$\begin{array}{c} E_{k}=\underset{\Omega }{\int }\frac{1}{2}\rho {\left|u\right|}^{2}\;d\Omega ,\; \end{array}$$

The gravitational potential energy (with reference to center of mass) is:

(14) $$\begin{array}{c} E_{grav}=\rho gV_{w}h \end{array}$$

where, h, is the height of the center of mass with reference to the solid substrate and, $V_{w}$, is the droplet’s volume.

The main advantage of our continuum level, sharp-interface modelling approach is the removal of the stress singularity near the TPL without the need of theoretical correlations, such as the Cox relation for the contact line movement. As a result, there is no need for defining empirical constants regarding the slip region around the contact line, but only the parameters of the solid–liquid interactions. The droplet detachment, if it occurs, will emerge naturally without the need of a manual detachment condition for the outer contact line. Furthermore, substrates with any kind of geometrical or chemical complexity, from macroscale to microscale, can be simulated in a unified manner and at the same time, the computational cost is kept very low. We report that, for an 2D axisymmetric simulation of a droplet jumping over a micro-structured surface, the typical size of the computational problem is of the order of $10^{4}$ degrees of freedom, which requires, approximately, a solution time of 2 hours, performed on a dual processor computer (with 2 Intel^®^ Xeon^®^ processors E5-2640 @2.50 GHz) using COMSOL Multiphysics^®^. Finally, the calculation of derived quantities, such as the energy dissipation, due to pinning of the multiple contact lines at the surface structures, is straightforward and there is no need for extensive theoretical modelling.

## 3. Results and Discussion

### 3.1. Validation of Computational Model

We first validate our model against the numerical results of Cavali et al. [18]. We consider an axisymmetric water droplet of density, $\rho_{w}=1000\;\mathrm{kg}/m^{3}$, viscosity, $\mu_{w}=1.0016\;\mathrm{mPa}\;s$ and volume, $V_{w}=5.3\;\mu L$, surrounded by an oil phase of density, $\rho_{o}=760\;\mathrm{kg}/m^{3}$, and viscosity, $\mu_{o}=0.494\;\mathrm{mPa}\;s$, on a flat solid substrate. The water-oil interfacial tension is, $\gamma_{wo}=24\;\mathrm{mN}/m$. The Young’s contact angle is, $\theta_{Y}=170^{{}^{\circ}}$ (voltage OFF), and an apparent contact angle of, $\theta_{0}=90^{{}^{\circ}}$ (voltage ON), is considered. The activation is achieved after instantly switching the contact angle from an initial value of $\theta_{0}$ to$\;\theta_{Y}$, as a result of switching the voltage OFF. The characteristic length scale is selected as $R_{0}={\left(\frac{3V_{w}}{4\pi }\right)}^{\frac{1}{3}}\approx 1.1\;\mathrm{mm}$ and the characteristic time, $T_{c}=\sqrt{\frac{R_{0}^{3}\rho_{w}}{\gamma_{wo}}}\approx 7\;\mathrm{ms}$.

**Figure 2:**
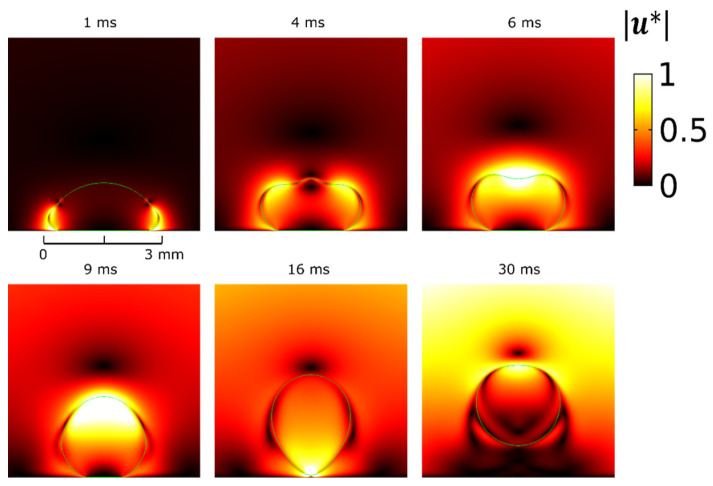
Time snapshots showing the dynamics during retraction, detachment and jumping of a 5.3 μL water droplet surrounded by oil as predicted by our numerical model. The dimensionless velocity magnitude $\left|u^{*}\right|$ is visualized as a colormap.

In Figure 2, we present selected time snapshots of a side view of the axisymmetric water droplet. The colormap corresponds to dimensionless velocity magnitude, $\left|u^{*}\;\right|=\frac{\left|u\right|}{u_{cap}}$, where $u_{cap}=\frac{R_{0}}{T_{c}}$. The results for the droplet shape deformation and the velocity field around the droplet, shown in Figure 2, are in good agreement with the corresponding snapshots in [18]. Furthermore, we calculate a maximum normalized height of the center of mass, $h_{max}^{*}=\frac{h_{max}}{R_{0}}\approx 2$, which agrees with the value shown in [18], for the same contact angle and droplet radius. Some discrepancies arise between numerical results in the time evolution; particularly our simulations give faster dynamics and an earlier time of droplet detachment. The detachment time is 16 ms compared to 17.5 ms shown in Figure 4a of [18]. This deviation could be attributed to the finite size of the domain used for bounding the oil liquid phase. Nevertheless, the model is capable of capturing the dynamics of retraction and detachment naturally, without the use of any explicit or manually-implemented critical detachment conditions.

One of the major advantages of our modelling approach as compared to the traditional hydrodynamics models is that any effect of dynamic contact angle hysteresis, can be predicted naturally by introducing a friction term (Navier slip) with a slip coefficient to recover no-slip boundary conditions. The effect of static hysteresis can also be predicted by the model for the case of a structured substrate, as explained in [23].

### 3.2. Micro-Structured Surfaces

In many industrial applications it is essential to perform droplet removal in ambient conditions. An alternative approach for enhancing the hydrophobicity (i.e., to achieve high contact angles by lowering the adhesion strength) without the use of a surrounding oil phase is to introduce geometrically micro-structured surfaces. A lower adhesive strength, could improve at the same time, the energy efficiency of the detachment/jumping process, as also suggested in [18]. We continue our investigation using our proposed numerical modelling approach, focusing on the effect of the micro-topography on the dynamics and the energy efficiency of the process, compared to a perfectly flat surface case, for a droplet in ambient conditions.

For simplicity we perform computations for an axisymmetric water droplet of volume $V_{w}=7.7\;\mu L$ ($R_{0}=1.225\;\mathrm{mm}$) and $\gamma_{wa}=72.8\;\mathrm{mN}/m$ (water–air). That corresponds to a capillary time of, $t_{c}\approx 5\;\mathrm{ms}$. The surrounding air medium is not taken into account in the simulations (negligible density and viscosity compared to water). We examine flat and structured surfaces with different types of axisymmetric stripes. The Young’s contact angle is stepwisely changed from $107^{{}^{\circ}}$ (corresponding to voltage ON) to $150^{{}^{\circ}}$ (voltage OFF). Such a high contact angle can be achieved by a secondary roughness scale (at the nanoscale) [32,33] which is not simulated but it is incorporated as a part of the material wettability properties. We use the same values of the Young’s contact angle for the flat surface in order to directly compare the results. Notice that the effective (macroscopic) contact angle in the case of the micro-structured surfaces will be higher due to the enhancement of hydrophobicity due to the structure.

**Figure 3:**
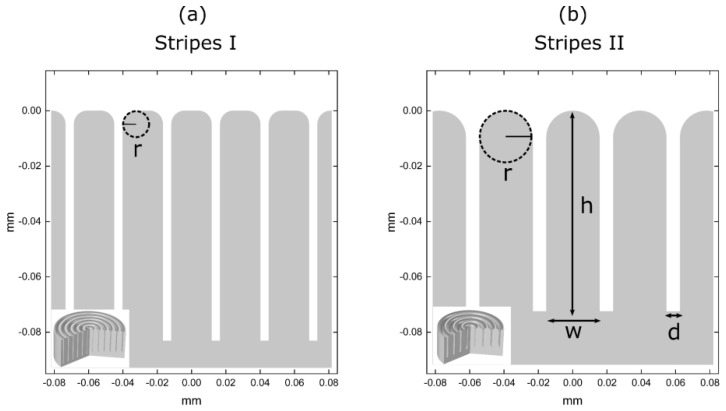
Cross-section and 3D slice of the 2 micro-structured surfaces used. (**a**) axisymmetric stripes with h=85 μm, w=15 μm, d=3 μm and r=5 μm and (**b**) axisymmetric stripes with h=75 μm, w=20 μm, d=5 μm and r=10 μm.

We study two representative cases of micro-structured surfaces: the first one (labeled as “Stripes I”), where the droplet detachment is found to be successful, and the second one (labeled as “Stripes II”), where the droplet detachment, as our computations showed, is unsuccessful. A detailed view of the structures and their geometrical characteristics is shown in Figure 3. The values of the effective (macroscopic) contact angle can be estimated theoretically by the Cassie–Baxter (CB) relation, assuming that the values of the Young’s contact angle are large enough:

(15) $$\begin{array}{c} cos\theta_{eff}=-1+\varphi_{s}\left(1+cos\theta_{Y}\right),\; \end{array}$$

where, $\varphi_{s}$ is the fraction of solid area which is in contact with the liquid, calculated by Equation (16):

(16) $$\begin{array}{c} \varphi_{s}=\frac{A_{LS}}{A_{S}},\; \end{array}$$

where, $A_{S}=A_{LA}+A_{LS}$ is the droplet footprint area.

In Table 1, we show the values of the effective contact angle for the two micro-structured surfaces (namely, Stripes I and Stripes II), calculated using Equation (15), as well as the ones obtained by our model. In the latter case the contact angle is computed by the slope of third order polynomial fitting to the computed droplet profile near the three-phase contact line. For the first micro-structured surface (“Stripes I”), the Equation (15) predicts an effective contact angle variation of $\Delta \theta_{eff}=39^{{}^{\circ}}$ whereas our model predicts a value of,$\;\Delta \theta_{eff}=33^{{}^{\circ}}$. Similarly, for the second micro-structured surface (“Stripes II”), the two methods predict a value of $\Delta \theta_{eff}=37^{{}^{\circ}}$ and, $\Delta \theta_{eff}=25^{{}^{\circ}}$, respectively. For a flat surface, $\theta_{eff}\equiv \theta_{Y}$. Both methods qualitatively agree that the second micro-structured surface is the most hydrophobic, due to a lower fraction of liquid–solid contact. However, the magnitude of the effective contact angle variation, calculated numerically, is significantly lower than the one calculated using Equation (15), especially for the second micro-structured surface. This deviation appears due to the smoothing of the stripe edges, controlled by the parameter, r, which is not introduced in the Equation (16) for the calculation of, $\varphi_{s}$. Moreover, for, $\theta_{Y}=107^{{}^{\circ}}$, the Cassie–Baxter relation is, somewhat, unreliable since the determination of, $\varphi_{s}$_,_ requires determination of the exact droplet profile, particularly, in what concerns the contact with the solid surface.

**Figure 4:**
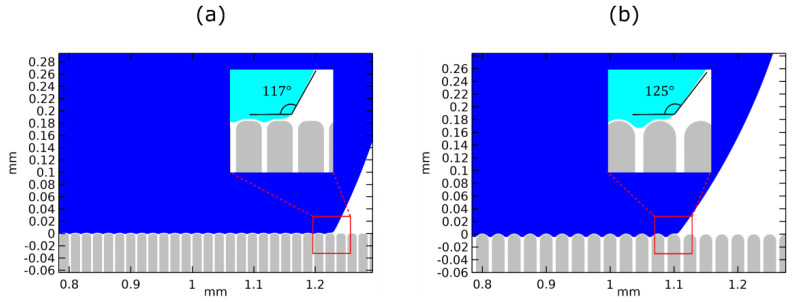
Magnification of the droplet equilibrating over surface asperities with a Young’s contact angle $\theta_{Y}=107^{{}^{\circ}}$ (equivalent to electrowetting actuated state) for (**a**) h=85 μm, w=15 μm, d=3 μm and r=5 μm with an effective contact angle of $117^{{}^{\circ}}$ and (**b**) d=75 μm, w=20 μm, d=5 μm and r=10 μm with an effective contact angle of $125^{{}^{\circ}}$.

A magnified view of the droplet equilibrating over surface asperities, before the restoration of the wettability (when voltage is ON), for both micro-structured substrates, showing the effective contact angles, can be found in Figure 4.

**Figure 5:**
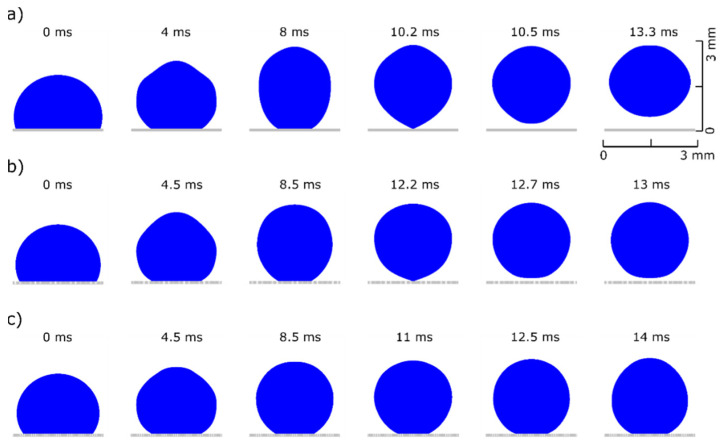
Water droplet dynamics in air medium over different topographies. (**a**) perfectly smooth surface where the droplet detaches at a time of 10.2 ms and the center of mass reaches at a maximum height of 1.49 mm (see Video S1 of the supplementary material), (**b**) surface with axisymmetric stripes of h=85 μm, w=15 μm, and where the droplet detaches at a later time of 12.2 ms and the center of mass reaches a smaller maximum height of 1.28 mm (see Video S2) and (**c**) surface with axisymmetric stripes of h=75 μm, w=20 μm, d=5 μm and r=10 μm where no detachment occurs (see Video S3).

In the next section, we present the droplet dynamics for the two micro-structured surfaces, and compare them with the corresponding dynamics of the droplet on the flat substrate. The proposed modelling approach allows for a detailed analysis of the de-wetting process. As seen in Figure 5, for the same Young’s contact angle variation, $\Delta \theta_{Y}=43^{{}^{\circ}}$, the droplet detaches earlier and jumps at a higher height in the case of the flat surface. In particular, the detachment time is $t_{d}=10.2\;\mathrm{ms}$ and the maximum height of the center of mass is $h_{max}=1.49\;\mathrm{mm}$, compared to $t_{d}=12.2\;\mathrm{ms}$ and $h_{max}=1.28\;\mathrm{mm}$ for the first structured surface (Figure 5b). In the case of the second structured surface, the droplet does not have enough excess surface energy to overcome the combination of the energy barrier due to surface adhesion and energy loses due to viscous dissipation. Therefore, the droplet cannot be detached from the surface. The droplet’s wetting state under electrowetting actuation (when voltage is ON) affects the efficiency of the excess surface energy conversion to kinetic energy required for jumping. As discussed above, introducing a structured topography on the solid surface can increase the hydrophobicity (smaller adhesion) by decreasing the effective contact area, but at a cost of lowering effective contact angle variation, increasing pinning effects or leading to a wetting transition from Cassie to Wenzel state (fully-wetted or collapsed state).

**Figure 6:**
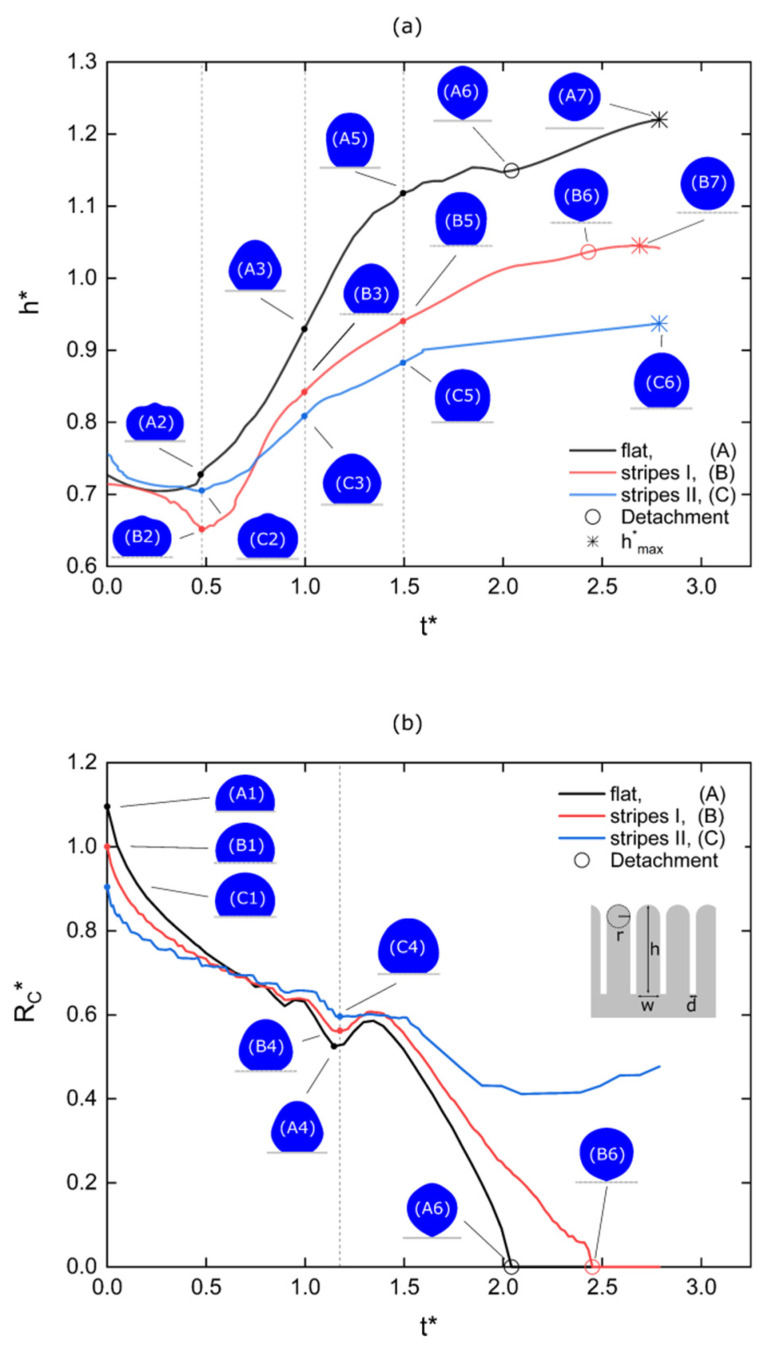
(**a**) time evolution of the normalized height (center of mass) for the case of the flat surface (black line, (A)), surface with axisymmetric stripes of h=85 μm, w=15 μm, d=3 μm and r=5 μm (red line, (B)) and surface with axisymmetric stripes of h=75 μm, w=20 μm, d=5 μm and r=10 μm (blue line, (C)). States (A6) and (B6) corresponds to detachment and (A7), (B7), (C6) to acquisition of maximum height. (**b**) time evolution of the normalized contact radius for the same cases as (a). States (A1), (B1), (C1) corresponds to initial contact radius before changing the wettability. For the perfectly smooth substrate the time of detachment is approximately $2t_{c}$ and for the first structured substrate is approximately $2.5t_{c}$.

A more detailed demonstration of the de-wetting/jumping dynamics can be seen in Figure 6. In Figure 6a we plot the normalized height of the center of mass. As reported in previous works [15,16,19], the droplet motion initiates at the contact line ($0\leq t^{*}\leq 0.5$). For all cases a global minimum height is achieved at $t^{*}\approx 0.5$ (states (A2), (B2), (C2)). The motion continues, located at the upper part of the droplet where the height increases monotonically ($0.5\leq t^{*}\leq 1.5$, states (A3), (B3), (C3) and (A5), (B5), (C5)). After that, the contact radius sharply decreases as the neck develops, breaks and the droplet detaches from the surface (states (A6) and (B6)). A maximum height is reached after jumping, and then an almost free spherical shape is formed (states (A7) and (B7)). Again, it is evident that for the case of the flat surface the jumping process is quantitatively more efficient: the droplet detaches earlier and reaches a higher maximum height.

**Figure 7:**
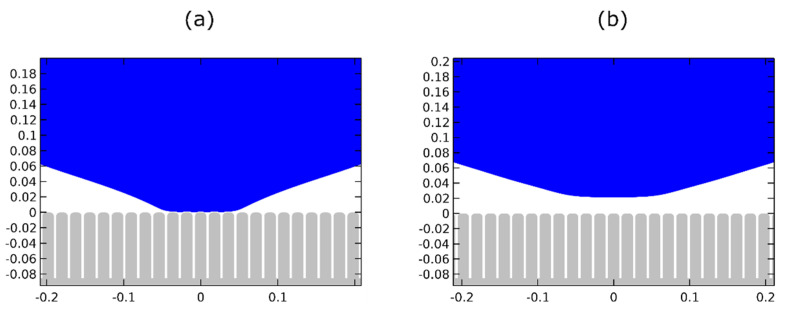
Magnification of the droplet (**a**) before and (**b**) after detachment from the micro-structured surface (stripes I), (h=85 μm, w=15 μm, d=3 μm and r=5 μm).

In Figure 6b the time evolution of the normalized contact radius, $R_{c}^{*}$, is presented. In particular, we consider that the contact radius is the position of the outer (or macroscopic) contact line which is defined as the intersection of the droplet surface with a horizontal baseline just above the substrate ($z\approx 3\times 10^{-3}R_{0}).$ At $t^{*}=0$ (states (A1), (B1), (C1)) the contact radius corresponds to the initial state before changing the wettability (i.e., before switching the voltage OFF). The values of the initial contact radius ($R_{c}^{*}\approx 1.1\;$for A, $R_{c}^{*}\approx 1\;$for B and $R_{c}^{*}\approx 0.9\;$for C) confirm that the adhesion decreases from A to C. The contact radius decreases as the droplet de-wets the solid surface. The de-wetting on the micro-structured surfaces is performed gradually, stripe by stripe, causing fluctuations in the contact radius evolution. However, for the first micro-structured surface (stripes I), at the moment of detachment, we notice a sudden de-wetting of several stripes (see Figure 7).

It is worth mentioning that at, $t^{*}\approx 1.2$ (states (A3), (B3), (C3)) a local minimum contact radius value is reached for all the three cases. This minimum value is related with a surface wave developed due to the stepwise modification of the wettability. The onset is close to the capillary time, which mainly depends on the fluid properties and volume, and appears independent of the surface topography. The detachment onset for the perfectly smooth substrate is, $t^{*}\approx 2$, and for the first structured substrate is, $t^{*}\approx 2.5$. We attribute this delay on the presence of the surface asperities which inhibit the liquid retraction.

The surface structure, as our computations showed, affects the droplet dynamics, such as the maximum jump height and the detachment time. This statement can be supported by computing the available excess energy and the energy dissipation for each case.

We compare the normalized available excess energies between all the three cases (see Equation (10)):

(17) $$\begin{array}{c} \Delta E_{ex}^{*}=\frac{\Delta {Ε}_{ex}}{\Delta E_{ex,\;A}},\; \end{array}$$

where $\Delta E_{ex,A}$ is the available excess energy of the droplet for the flat solid substrate. We calculate a value, $\Delta E_{ex,B}^{*}\approx 0.49$, for the first (stripes I) and a value, $\Delta E_{ex,C}^{*}\approx 0.39$, for the second micro-structured surface (stripes II); both of them are smaller than unity. Therefore, in both cases of micro-structures, the available excess energy is lower than the case of the flat surface. Furthermore, the non-detachable droplet has the lowest available excess energy: that is $\frac{1-0.39}{1}100\%\approx \;61\%\;$lower than the detachable droplet from the flat surface and $\frac{0.49-0.39}{0.49}100\%\approx \;20\%\;$lower than the detachable droplet from the first micro-structured surface. The available excess energy is strongly associated with the macroscopic (effective) contact angle variation and not the microscopic contact angle variation (which is the same for both three cases). In a similar manner, the numerically calculated effective contact angle variation, normalized with respect to the contact angle variation of the flat substrate, is $\Delta \theta_{eff,B}^{*}\approx 0.77$ for the first and $\Delta \theta_{eff,B}^{*}\approx 0.58$ for the second micro-structured surface. The non-detachable droplet has the lowest effective contact angle variation: that is $\frac{1-0.58}{1}100\%\approx \;42\%\;$lower than the detachable droplet from the flat surface and $\frac{0.77-0.58}{0.77}100\%\approx \;24\%\;$lower than the detachable droplet from the first micro-structured surface. The lower available excess energy, due to the lower effective contact angle variation, is the main reason for not achieving droplet detachment in the case of the second micro-structure topography. Thus, one could, by performing more systematic computations, determine the critical contact angle modification for achieving detachment. Here we just demonstrate the ability of our model to simulate the detachment from structured surfaces.

Another mechanism affecting efficiency is the pinning of the multiple contact lines on the substrate asperities which leads to increased energy dissipation. We can quantify the energy loses due to contact line pinning by calculating the total normalized energy dissipation $E_{d}^{*}$ (due to viscous dissipation). This calculation is feasible not only for the entire droplet volume but can also be focused at the multiple contact lines, as an advantage of the modelling method used. The energy dissipation is normalized with the corresponding available excess energy, $E_{d}^{*}=\frac{E_{d}}{\Delta {Ε}_{ex}}$. For the normalized total (total by means of volume and time) dissipation we calculate a value of $E_{d,\;A}^{*}\approx 0.14$ for the flat, a value of $E_{d,\;B}^{*}\approx 0.22$ for the first and a value of $E_{d,\;C}^{*}\approx 0.81$ for the second micro-structured surface. It is clear that a significantly larger fraction of excess surface energy for the non-detachable droplet is dissipated.

The results concerning the available excess energies and the energy dissipation support the observations in Figure 5 and Figure 6 for the maximum height obtained in each case. The droplet over the flat substrate (A) has the highest available excess energy, dissipates the lowest energy fraction and, as a result, obtains the highest maximum height. In a same manner, the droplet over the second micro-structured surface (stripes II or C) has the lowest available excess energy, dissipates the highest energy fraction and, consequently, cannot even detach from the surface.

Finally, we discuss the energy efficiency of the process for the flat and the first micro-structured surface, where jumping occurs. For simplicity, we define the energy efficiency as the fraction of available excess energy which is converted to gravitational potential energy gain:

(18) $$\begin{array}{c} n_{eff}=\frac{E_{g,max}-E_{g,0}\;}{\Delta {Ε}_{ex}},\; \end{array}$$

where $E_{g,max}$ is the gravitational potential energy at the maximum height and $E_{g,0}$ is the initial gravitational potential energy. The efficiency for the flat surface is $n_{eff,A}\approx 41\%$ and for the first micro-structured surface is $n_{eff,B}\approx 55\%$. Although the jumping process is more effective in the case of the flat substrate, due to higher available excess energy, the overall energy efficiency (i.e., the conversion of the excess energy to kinetic/gravitational energy) is higher for the case of the first micro-structured substrate. As a result, in contrast to the drawbacks discussed above, such as reduction of available excess energy and higher energy dissipation due to pinning effects, the use of micro-structured substrates seems to improve the overall energy efficiency of the detachment/jumping process if a suitable surface structure is selected. The limit of this improvement is out of the scope of this study, and remains to be explored. In such study one should also take into account the effect of the contact angle reversibility when voltage is switch between ON and OFF. A comprehensive study on the reversibility of electrowetting on structured surfaces can be found in Kavousanakis et al. [33] and Papathanasiou [34].

Geometric parameters, such as height, width, density and smoothness of surface structures could be controlled and optimized, using the energy efficiency (Equation (18)) as an objective function, in order to achieve droplet removal with the lowest possible contact angle variations and consequently, the lowest energy consumption during EW actuation.

## 4. Conclusions

In this work we presented the capabilities of a recently developed modelling approach by our group, in simulating, probably for the first time, electrowetting-induced droplet detachment and jumping over topographically micro-structured surfaces. We validated our model against the work of Cavali et al. [18] and performed computations for two representative cases of micro-structured surfaces in order to show that we can capture, in detail, the retraction dynamics and jumping and quantify the role of adhesion and energy dissipation due to pinning on the surface asperities. The outcome of this numerical study reveals a connection between surface micro-structure and energy efficiency of the droplet detachment and jumping process. We strongly believe that further numerical simulations using this modelling approach, could enable the proper design of surfaces, in order to achieve the highest energy efficiency during droplet removal under electrowetting actuation. This could be achieved by properly designing the surface structures in order to minimize the critical contact angle modification required for droplet detachment. Another possible area of future research would be the coupling of the modified hydrodynamics equations with the equations of electrostatics in order to predict the required voltage for detachment from micro-textured surfaces.

## Figures and Tables

**Table 1:** Effective contact angles at the two wetting states (voltage ON and OFF) and contact angle modification, for the flat and the two micro-structured surfaces. The effective contact angles are computed using third order polynomial fitting and the Cassie–Baxter relation (the values inside parentheses).

	Flat	Stripes I	Stripes II
	$\theta_{eff}\equiv \theta_{Y}$	$\theta_{eff}$	$\theta_{eff}$
Voltage ON	$107^{{}^{\circ}}\;$	$117^{{}^{\circ}}\;\left(114^{{}^{\circ}}\right)$	$125^{{}^{\circ}}\;\left(116^{{}^{\circ}}\right)$
Voltage OFF	$150^{{}^{\circ}}$	$150^{{}^{\circ}}\;\left(153^{{}^{\circ}}\right)$	$150^{{}^{\circ}}\;\left(153^{{}^{\circ}}\right)$
$\Delta \theta_{eff}$	$43^{{}^{\circ}}$	$33^{{}^{\circ}}\;\left(39^{{}^{\circ}}\right)$	$25^{{}^{\circ}}\;\left(37^{{}^{\circ}}\right)$

## Supplementary Materials

The following are available online at https://www.mdpi.com/article/10.3390/mi12060592/s1, Video S1: Droplet dynamics for the flat substrate (Figure 5a). Video S2: Droplet dynamics for the first micro-structured surface (Stripes I, Figure 5b). Video S3: Droplet dynamics for the second micro-structured surface (Stripes II, Figure 5c).
